# SARS-CoV-2 and Epstein–Barr Virus-like Particles Associate and Fuse with Extracellular Vesicles in Virus Neutralization Tests

**DOI:** 10.3390/biomedicines11112892

**Published:** 2023-10-25

**Authors:** Johannes Roessler, Dagmar Pich, Verena Krähling, Stephan Becker, Oliver T. Keppler, Reinhard Zeidler, Wolfgang Hammerschmidt

**Affiliations:** 1 Department of Otorhinolaryngology, University Hospital, Ludwig-Maximilians-Universität (LMU) München, 81377 Munich, Germany; johannes.roessler@helmholtz-munich.de (J.R.); reinhard.zeidler@helmholtz-munich.de (R.Z.); 2 Research Unit Gene Vectors, Helmholtz Zentrum München, German Research Center for Environmental Health, 81377 Munich, Germany; dagmar.pich@helmholtz-munich.de; 3 German Centre for Infection Research (DZIF), Partner Site Munich, 81377 Munich, Germany; keppler@mvp.lmu.de; 4 Institute of Virology, Faculty of Medicine, Philipps University Marburg, 35043 Marburg, Germany; kraehliv@staff.uni-marburg.de (V.K.); becker@staff.uni-marburg.de (S.B.); 5 German Centre for Infection Research (DZIF), Partner Site Giessen-Marburg-Langen, 35043 Marburg, Germany; 6 COVID-19 Registry of the LMU Munich (CORKUM), LMU University Hospital, 81377 Munich, Germany; 7 Max von Pettenkofer Institute and Gene Center, Virology, National Reference Center for Retroviruses, Faculty of Medicine, Ludwig-Maximilians-Universität (LMU) München, 81377 Munich, Germany; 8 Institute of Structural Biology, Helmholtz Munich, 85764 Neuherberg, Germany

**Keywords:** SARS-CoV-2, virus-like particle, EBV, Epstein–Barr virus, virus neutralization test, antibody, diagnostic test, extracellular vesicle, fusion

## Abstract

The successful development of effective viral vaccines depends on well-known correlates of protection, high immunogenicity, acceptable safety criteria, low reactogenicity, and well-designed immune monitoring and serology. Virus-neutralizing antibodies are often a good correlate of protective immunity, and their serum concentration is a key parameter during the pre-clinical and clinical testing of vaccine candidates. Viruses are inherently infectious and potentially harmful, but we and others developed replication-defective SARS-CoV-2 virus-like-particles (VLPs) as surrogates for infection to quantitate neutralizing antibodies with appropriate target cells using a split enzyme-based approach. Here, we show that SARS-CoV-2 and Epstein–Barr virus (EBV)-derived VLPs associate and fuse with extracellular vesicles in a highly specific manner, mediated by the respective viral fusion proteins and their corresponding host receptors. We highlight the capacity of virus-neutralizing antibodies to interfere with this interaction and demonstrate a potent application using this technology. To overcome the common limitations of most virus neutralization tests, we developed a quick in vitro diagnostic assay based on the fusion of SARS-CoV-2 VLPs with susceptible vesicles to quantitate neutralizing antibodies without the need for infectious viruses or living cells. We validated this method by testing a set of COVID-19 patient serum samples, correlated the results with those of a conventional test, and found good sensitivity and specificity. Furthermore, we demonstrate that this serological assay can be adapted to a human herpesvirus, EBV, and possibly other enveloped viruses.

## 1. Introduction

Vaccines are among the most successful and efficient means to counteract infectious diseases. Prophylactic immunizations effectively prevent millions of deaths and serious diseases worldwide every year and substantially contributed to the eradication of harmful viruses in the past [1]. Vaccines often provide effective protection against infectious viral pathogens, for which therapeutic options are limited. Immunizations by natural infection or vaccination induce adaptive cellular and humoral immune responses that ideally result in sustained immune memory [2]. Neutralizing antibodies (NAbs) can have a key role as these immunoglobulins can prevent the initial or re-infection of cells and thereby limit viral spread in the host by blocking the cell entry of viruses. A prominent example emerged from the coronavirus disease 2019 (COVID-19) pandemic when the serum titer of specific NAbs was identified as a relevant diagnostic parameter and correlate of protection from severe disease [3,4,5]. Moreover, NAb titers are important in evaluating the immunogenicity and efficacy of vaccine candidates in clinical development, but their quantitation by virus neutralization tests (VNTs) can be problematic with certain viruses.

Conventional VNTs (cVNTs) rely on replication-competent, infectious viruses, permissive target cells and often on in vitro cytopathic effects (CPEs) that appear as foci or plaques several days after infection, reflecting the number of infectious viruses. Depending on the individual virus, CPE counting can be cumbersome or even impossible without additional methods, such as immunostaining, limiting dilution approaches, or PCR, to quantify viral infection [6]. All cVNTs have to meet biological safety levels (BSLs) to handle infectious agents. Partial workarounds are pseudotyped VNTs (pVNTs), which rely on replication-deficient viral vectors that can be handled at lower BSLs and are equipped with a phenotypic marker. Marker readouts are often based on the de novo transcription and translation of a reporter gene encoding, e.g., green fluorescence protein. pVNTs are widely used, but the composition and infectivity of such pseudotyped retro-, lenti-, or rhabdoviral vector particles can differ from the original virions [6]. Surrogate VNTs (sVNTs) are mostly based on immobilized recombinant protein domains and do not quantitate neutralization as such but detect antibodies that interfere with protein–protein, i.e., receptor–ligand, interactions. Therefore, their interpretation can suffer from weak correlations to cVNTs [7,8,9].

The severe acute respiratory syndrome coronavirus 2 (SARS-CoV-2), which caused the most recent pandemic, is an enveloped virus and a member of the family *Coronaviridae*. SARS-CoV-2 virions contain a positive-sense, single-stranded RNA genome of almost 30 kb, which codes for four structural proteins, spike (S), envelope (E), membrane (M), and nucleoprotein (N), and several accessory proteins, which have important roles in viral replication or immune modulation [10,11]. The homotrimeric glycoprotein S consists of two subdomains S1 and S2, which are separated by a furin cleavage site and contain the receptor-binding domain (RBD) and the fusion peptide, respectively [12]. During viral egress, S is often proteolytically processed at the S1/S2 site, which facilitates a second cleavage at the S2′ site within the S2 domain during entry [13,14]. On host cells, angiotensin-converting enzyme 2 (ACE2) acts as a receptor for S. After attachment, virus entry into susceptible cells occurs either via fusion with the plasma membrane and S2′ cleavage by the cell surface protease TMPRSS2 [15] or via endocytosis and processing by cathepsin L (CTSL) [13]. The cytoplasmic release of the genomic viral RNA then initiates the viral replication cycle [11]. NAbs against SARS-CoV-2, either acquired by convalescence or vaccination, have been shown to directly correlate with protection from severe COVID-19 [3,4,5]. As another human pathogen, the Epstein–Barr virus (EBV) is a complex, enveloped, double-stranded DNA virus of the *Herpesviridae* family (human gammaherpesvirus 4, HHV-4) that targets mature B cells very efficiently. EBV is an oncogenic virus, which establishes lifelong persistent infection. It can induce an acute viral disease, infectious mononucleosis, and is associated with various types of cancers and autoimmune diseases, such as multiple sclerosis [16,17,18]. EBV virions are decorated with 12 viral glycoproteins, among them gp350, gp42, gH, gL, and the fusion protein gB. No marketed EBV vaccine is available, but vaccine candidates that are currently being developed require thorough immune monitoring and diagnostics, including a simple and reliable VNT, which is not readily available [16,17].

Extracellular vesicles (EVs), which are naturally released from cells, are structurally similar to enveloped viruses as both are membranous vesicles, but EVs do not replicate or reprogram cells. EVs vary in size from 50 to 1000 nm in diameter and consist of proteins, lipids, RNA, DNA, and other constituents of the cell from which they originate. Because of their ubiquity in body fluids and their suggested role in intercellular communication, EVs are considered promising circulating biomarkers for various diseases and tools for novel therapies [19]. Compared to enveloped viruses, which are equipped with dedicated glycoproteins to engage with cellular receptors and mediate fusion with their target cells [20,21], EVs do not enter cells productively [22].

Until recently, little was known about EV–EV or virus–EV fusion. Induced by cellular components and acidification, the fusion of EVs with large unilamellar vesicles has been demonstrated [23]. The direct fusion of virions with liposomes has been shown before via lipid dye fluorescence dequenching for Zika virus with isolated and immobilized vesicles on a solid surface, but artificially triggered by complementary DNA strands tethered to the membranes [24,25]. The entry of influenza virus was mimicked by the fusion of immobilized membranes via hemagglutinin and sialic acid and induced by acidification [26]. For SARS-CoV-2, virus–EV fusion was suggested as ACE2 EVs were shown to impair infection with pseudotyped viral vector particles in vitro [27] and later observed with immobilized vesicles by fluorescence dequenching [28].

In contrast to inherently infectious and potentially harmful viruses, virus-like particles (VLPs) are devoid of viral DNA or RNA and therefore cannot replicate. Therefore, VLPs are considered attractive surrogates for basic research and vaccine candidates. VLPs can be obtained from cells that express and assemble one or several viral structural proteins and incorporate them into nascent EVs. Recently, we and others developed such VLPs of SARS-CoV-2 and demonstrated entry into susceptible cells [29,30,31]. Back-to-back with Kumar et al. (2021), we used the identical split enzyme approach to trace the VLPs and complementing cell-based entry models, but we developed a quick and easy VNT based on this principle that we characterized in depth [29]. Using SARS-CoV-2 VLPs loaded with the ‘HiBiT’ part of the split nanoluciferase [32], cell-free VLP–EV fusion was successfully shown with EVs that bear the ACE2 receptor and the second part of the split nanoluciferase, ‘LgBiT’ [33]. This model was utilized to study the fusion and inhibition mechanisms of S protein domains [34,35].

We now extend this principle and describe a second generation of an advanced, cell-free VNT. VLPs co-associate and fuse with EVs derived from the virus’ target cells following virus-specific tropism. We characterized this interaction in detail using biochemical, physical, and functional techniques and demonstrated that virus-specific NAbs block VLP–EV interaction and fusion. A quick and quantitative VNT was established, which is completely independent of infectious viruses, genetically modified organisms, and importantly, living cells. We compare this cell-free VLP neutralization test (cfVLPNT) with a cVNT on a large set of double-blinded COVID-19 patient serum samples. Moreover, we show that VLP–EV association and fusion are not limited to SARS-CoV-2 but can be extended to an unrelated human pathogen: the Epstein–Barr virus. We propose that cfVLPNTs are a feasible and generally applicable technique for designing rapid, quantitative, scalable neutralization tests for enveloped viruses under the conditions of biosafety level 1.

## 2. Material and Methods

### 2.1. Cell Lines and Cell Culture

HEK293T (CID3915), U251MG, hACE2^+/−^ (CID4663, CID4629), U251MG, hACE2^+/−^, NM∼LgBiT^+^ (CID4697, CID4685), U251MG, hACE2^+/−^, CD63∼LgBiT^+^ (CID4727, CID4694), HEK293, CD63∼LgBiT^+^, EB-VLP producer (CID4797), and Daudi, CD63∼LgBiT^+^ (CID4821) cells were maintained in DMEM (Gibco, Thermo Fisher Scientific, Waltham, MA, USA) and supplemented with 8% FBS (AC-SM-0143, Anprotec), penicillin (Pen), and streptomycin (Strep) (Gibco, Thermo Fisher Scientific). Cell lines were cultivated at 37 °C in a water-saturated atmosphere with 5% CO_2_. Cell viability was monitored via trypan blue exclusion, and cultures with more than 95% viable cells were used in all experiments. Cell lines constitutively expressing hACE2, NM∼LgBiT, CD63∼LgBiT, or CD63∼HiBiT were generated via the retroviral transduction or transient transfection of parental cells with expression plasmids followed by selection with the respective antibiotic, single-cell seeding or flow cytometric cell sorting.

### 2.2. Generation and Purification of SARS-CoV-2- and EBV-like Particles and Engineered EVs

To generate the S^+^ VLP of SARS-CoV-2 S (B.1, B.1.617.2 or B.1.1.529 BA.1) and ∆S VLP, HEK293T cells were seeded and transiently transfected the next day, with carefully adjusted ratios of five codon-optimized expression vectors coding for M, N, E, and S or mock, together with CD63∼HiBiT as described by Roessler et al. [29] The expression plasmids are termed p7413.1 (S:B.1), p7487.IA1 (S:B.1.617.2), p7501.EA20 (S:B.1.1.529 BA.1), p7519.KA1 (S^∆Furin^: D614G, R682G, R683G, R685G), p5025 (mock), p7447.SA11 (CD63∼HiBiT), p7395.LA3 (M), p7396.NA9 (E), and p7391.MA5 (N). After 72 h, S^+^ VLP or ∆S VLP were harvested from conditioned cell culture supernatants (DMEM, 8% FBS, supplemented with Pen/Strep, Gibco, Thermo Fisher Scientific), centrifuged at low speeds at 4 °C for 10 min at 300× *g* and 20 min at 4200× *g*, and generally used without further processing. For hACE2^+/−^, CD63∼LgBiT^+^ EVs, 4 × 10^6^ U251MG (CID4727 or CID4694) were seeded on a 130 mm cell culture dish (168381, Thermo Fisher Scientific) in 18 mL DMEM (8% FBS, Pen/Strep) and incubated for 72 h prior to harvest from the conditioned media as described [29].

For EB-VLP, a CD63∼HiBiT^+^ HEK293 derivative (CID4797) of the ‘9G10′ cell line was induced with tamoxifen to translocate BZLF1 to the nucleus, inducing EBV’s lytic phase and secreting EB-VLP with several deleted or functionally inactivated viral genes, as described previously [36,37], prior to the harvest of the supernatant. B-lymphoblast-derived EVs were generated by seeding 7.5 × 10^5^ mL^−1^ CD63∼LgBiT^+^ Daudi cells (CID4821) in DMEM (8% FBS, Pen/Strep), cultivation at 37 °C for 96 h and harvest of conditioned media.

If indicated, S^+^ VLP, ACE2^+^ EV, EB-VLP, and Daudi EV supernatants were concentrated and further purified for some experiments. Sedimentation was achieved via ultracentrifugation at 100,000× *g*, 4 °C, and 2 h. Further separation was achieved via floatation and fractionation in a carefully stacked discontinuous iodixanol (Optiprep, Sigma-Aldrich, St. Louis, MI, USA) density gradient and ultracentrifugation at 160,000× *g*, 4 °C for 16 h, as described previously [29]. Particle fractions were flash frozen in liquid nitrogen for storage at −80 °C and subsequently characterized by WB, NTA, nanoflow technology, ELISA, Pierce BCA protein assay (23225, Thermo Fisher Scientific), and cellular- and cell-free VLP fusion assays.

### 2.3. Western Blot

Fractions of S^+^ VLP and ACE2^+^ EV were mixed with reducing- or non-reducing 5× Laemmli buffers (10% (*w*/*v*) SDS, 300 mM Tris/HCl pH 6.8, 30 vol% glycerol, and +/−25 vol% 2-mercaptoethanol) and incubated at 95 °C for 5 min if indicated. Samples were separated via 4–12% or 10% SDS-PAGE at 100 V in Bis-Tris-glycine buffers (MOPS NuPAGE, Thermo Fisher Scientific). Proteins were transferred to nitrocellulose membranes (GE Healthcare Life Science, Marlborough, MA, USA) via semi-dry blotting (Bio-Rad, Hercules, CA, USA) and blocked for 1 h in 5% (*w*/*v*) non-fat milk (Carl Roth, Wuerttemberg, Germany) in ddH_2_O at RT. Membranes were incubated at 4 °C overnight with primary antibodies (43A11, rat IgG2b, COVEV Helmholtz Zentrum München, München, Germany; GTX632604, mouse IgG, GeneTex, Irvine, CA, USA; PA5-81795, polyclonal rabbit antibody, Invitrogen, Waltham, MA, USA; 30E5, mouse IgG, Promega, Madison, WI, USA; N7100, mouse IgG, Promega) at 1:1000 or 1:2000 dilution in 5% (*w*/*v*) non-fat milk in ddH_2_O, washed 3 times in TBST (Tris-buffered saline with 0.1% Tween-20), and incubated at RT for 1 h with an HRP-conjugated secondary antibody (1112-035-062, goat anti-rat IgG, Jackson Immuno Research Europe, Cambridge, UK; 7076S, horse anti-mouse IgG, Cell Signaling, Danvers, MA, USA; 7074S, goat anti-rabbit IgG, Cell Signaling) at 1:20,000, 1:2000 and 1:2000 dilution, respectively, in 5% (*w*/*v*) non-fat milk in PBS. After 3× washing in TBST, blots were incubated with an ECL reagent (GE Healthcare) and imaged using Fusion FX (Vilber, Eberhardzell, Germany).

### 2.4. ELISA

The quantification of spike proteins in the fractions of S^+^ VLP was carried out using a sandwich enzyme-linked immunosorbent assay (ELISA) and two in-house generated anti-spike antibodies (43A11 and 55E10, COVEV Helmholtz Zentrum München) with non-overlapping epitopes following the previously described protocol [29]. For the calibration of the assay, recombinant spike protein (40589-V08B1, Sino Biological, Beijing, China) was used as a reference. Absorbance was measured using CLARIOstar Plus (BMG Labtech, Ortenberg, Germany), and data analysis was performed with GraphPad Prism 9.2.

### 2.5. NTA Analysis

Nanoparticle tracking analysis (NTA) was performed with the ZetaView PMX110 instrument (Particle Metrix) and the corresponding software (ZetaView 8.04.02) to measure the number and size distribution of VLP and EV preparations, as described previously [29].

### 2.6. Nano Flow Analysis of VLPs and EVs

VLP and EV preparations were analyzed using a CytoFLEX LX cytometer (Beckman Coulter Life Science, Indianapolis, IN, USA) as described previously [29]. Purified samples of S^+^ VLP, ∆S VLP, ACE2^+/−^ EV, EB-VLP, and Daudi EV were pre-stained with CellTraceViolet (CTV, C34571, Thermo Fisher Scientific) or CellTraceYellow (CTY, C34573, Thermo Fisher Scientific) at 37 °C for 20 min, quenched with 1% (*w*/*v*) bovine serum albumin (BSA) in PBS, and subjected to ultracentrifugation at 100,000× *g*, 4 °C, and 2 h, and the pellets were resuspended. Samples were normalized via dilution with PBS to equal particle density according to NTA data. Single samples or binary 1:1 combinations of VLPs and EVs were immediately diluted and subjected to flow cytometry (t_0_) or incubated at 37 °C for 16 h prior to analysis. Data were pre-gated according to their CTV, CTY fluorescence, and SSC-H signals before being subjected to Boolean ‘or’ gating. Combinations of samples were then analyzed for CTV^+^ CTY^+^ double-positive events. The antibody-mediated inhibition of co-association was performed via the pre-incubation of S^+^ VLP with 100 µg mL^−1^ of NmAb 35B12 (COVEV, Helmholtz Zentrum München) or isotype control 4H3 at 37 °C for 30 min before the addition of ACE2^+^ EV to a final antibody concentration of 30 µg mL^−1^ and incubation for 16 h at 37 °C. For antigen staining, pre-stained CTV^+^ EB-VLPs were incubated with anti-gp350 antibody 6G4 (Helmholtz Zentrum München) conjugated to AlexaFluor488 and diluted; afterwards, they were analyzed in the flow cytometer.

### 2.7. VLP Neutralization Test (VLPNT) for SARS-CoV-2

VLPTNs were conducted following the recently described protocol [29]. Therefore, 2 × 10^4^ U251MG (hACE2^+^, NM∼LgBiT^+^) cells (CID4697) in 100 µL DMEM (8% FBS, Pen/Strep supplemented) were seeded per well in a 96-well Lumitrac200 (655075, Greiner Bio-One, Munster, Austria) plate and incubated at 37 °C, overnight. On the following day, the serial dilutions of serum samples were prepared. Each of the 5× serum dilutions was added to normalized S^+^ VLP (S, M, N, E, and CD63∼HiBiT) to obtain a series of final dilutions ranging from 1:10 to 1:1801. S^+^ VLP and serum mixtures were incubated at 37 °C for 30 min, 50 µL was transferred to recipient cells after media removal, and cells were subsequently incubated at 37 °C for 4 h. Prior to the readout, the supernatant was removed and replaced with a 25 µL substrate mix (20 µL OptiMEM, Gibco, Thermo Fisher Scientific, +5 µL nano-Glo diluted 1:20 in LCS buffer, N2012, Promega). Luminescence was immediately quantified using a CLARIOstar Plus reader (BMG Labtech). The mean luminescence level of S^+^ VLP, with PBS instead of serum, was set to 0% neutralization while background luminescence obtained with ∆S VLP was set to 100% neutralization. For serum or antibody samples, titers that corresponded to 50% neutralization were determined via modeling nonlinear, sigmoidal 4PL curves using Graphpad Prism 9.3.1 and values were calculated, corresponding to 50% absolute signal reduction after background corrections. This value was termed ‘50% VLP neutralization titer’ (VLPN_50_). The WHO international standard (NIBSC 20/136) was used as in-between runs and as a within-run reference.

### 2.8. Cell-Free VLPNT (cfVLPNT) for SARS-CoV-2

For a cfVLPNT, serial dilutions of serum samples by a factor of 2.1 starting at 1:6 to 1:1081 were prepared via PBS in a 96-well plate. In total, 7 μL of each serum dilution was added to 28 μL of a normalized, 1:10 diluted S^+^ VLP (S, M, N, E, CD63∼HiBiT) supernatant in a 96-well plate to obtain a series of final dilutions ranging from 1:30 to 1:5403. In parallel, 5 μL of the serial serum dilution was added to 20 μL of ACE2^+^, CD63∼LgBiT^+^ EV supernatant in a 96-well Lumitrac200 (655075, Greiner Bio-One) to obtain equal final serum dilutions. Both mixtures were pre-incubated at 37 °C for 30 min before transferring 25 μL of the S^+^ VLP-serum mix to 25 μL of the ACE2^+^ EV–serum mix. The final mixture was then incubated at 37 °C for 4 h. Prior to the readout, 6.6 μL of nano-Glo diluted 1:20 in an LCS buffer (N2012, Promega) was added, and luminescence was immediately quantified in a CLARIOstar Plus reader (BMG Labtech). The VLPN_50_ calculation of the cfVLPNT was performed similarly as described for the VLPNT. For inhibitor experiments, S^+^ VLP and ACE2^+^ EV were pre-incubated separately for 30 min at 37 °C with chloroquine (C6628, Sigma-Aldrich) in ddH_2_O; camostat-mesylate (SML0057, Sigma-Aldrich) in DMSO; and BB94 batimastat (A2577, APExBIO, Houston, TX, USA) in DMSO, PBS, or DMSO prior to mixing with VLP and EV and incubation.

### 2.9. SARS-CoV-2 Neutralization Test (VNT_100_)

Human sera were heat-inactivated at 56 °C for 30 min and diluted in a series of two-fold dilution steps (1:4 to 1:512). One hundred plaque-forming units (PFUs) of SARS-CoV-2 stock (German isolate BavPat1/2020; European Virus Archive Global # 026 V-03883, GenBank: MZ558051.1) contained in 50 μL were added to an equal volume of diluted serum. The mixture was incubated at 37 °C, and approximately 2 × 10^4^ Vero C1008 cells (ATCC, Cat#CRL-1586) were added after 1 h. After a four-day incubation period, the cytopathic effect (CPE) was evaluated via light microscopy. Virus neutralization was defined as the complete absence of CPE in a given serum dilution (VNT_100_). The reciprocal geometric mean titer (GMT) was calculated from the highest serum dilution without CPE based on three replicates. The lower detection limit of the assay was 1:8, corresponding to the first dilution of the tested serum. Two positive controls were used as inter-assay neutralization standards and quality control for each test. The WHO international standard (NIBSC 20/136) was tested seven times, resulting in a GMT of 377 for a 100% absence of CPE in this test. Neutralization assays were performed in the BSL-4 laboratory of the Institute of Virology at Philipps University Marburg, Germany [29,38].

### 2.10. EBV Neutralization Test

For an EB-VLP-based VNT, human serum samples (EBV^+/−^ serostatus) or NmAbs (anti-gp42, 6H9; anti-gp350, 6G4; Helmholtz Zentrum München) were added to CD63∼HiBiT^+^ EB-VLP supernatants at 1:50 serum dilution or 10 µg mL^−1^ antibody concentration, respectively, and incubated for 30 min at 37 °C. Next, 6 × 10^4^ CD63∼LgBiT^+^ Daudi cells (CID4821) per well were pelleted via centrifugation in a 96-well Lumitrac200 (655075, Greiner Bio-One) plate, and the media were removed by pipetting. Subsequently, 50 µL of the EB-VLP mix was transferred to recipient cells and incubated at 37 °C for 4 h. Prior to readouts, supernatants were removed and replaced with a 25 µL substrate mix (20 µL OptiMEM, Gibco, Thermo Fisher Scientific, +5 µL nano-Glo diluted 1:20 in LCS buffer, N2012, Promega). Luminescence was immediately quantified in a CLARIOstar Plus reader (BMG Labtech).

### 2.11. Cell-Free EBV Neutralization Test

For the cell-free EB-VLP-based VNT, human serum samples (EBV^+/−^ serostatus) or NmAbs (anti-gp42, 6H9; anti-gp350, 6G4; Helmholtz Zentrum München) were added to the CD63∼HiBiT^+^ EB-VLP supernatant, and the purified CD63∼LgBiT^+^ Daudi EV fraction F3 separately, at 1:50 serum dilution or 10 µg mL^−1^ antibody concentration, respectively. After pre-incubation for 30 min at 37 °C, 25 µL of the EB-VLP mix and 25 µL of the Daudi EV mix were combined in a 96-well Lumitrac200 (655075, Greiner Bio-One) plate and incubated at 37 °C for additional 4 h. Prior to readout, 6.6 μL of nano-Glo diluted 1:20 in the LCS buffer (N2012, Promega) was added, and luminescence was quantified immediately using a CLARIOstar Plus reader (BMG Labtech).

### 2.12. Statistical Analysis

Data and statistical analyses were performed using Prism 9.3.1 (GraphPad Software, La Jolla, CA, USA). For main and supplementary figures, arithmetic mean values are displayed. Error bars for the y-axes indicate the standard deviation (SD) of at least three replicates or more if indicated. Prior to *t*-tests and one-way ANOVA, normal or log_10_-normal distribution according to Gaussian distribution was assured.

### 2.13. Patients and Serum Specimens

In this study, 57 COVID-19 serum specimens collected between 7 April 2020 and 29 December 2020 from 36 patients infected with SARS-CoV-2 and hospitalized at the LMU Klinikum, Munich, Germany, were included. All patients tested positive for SARS-CoV-2 via RT-qPCR in nasopharyngeal or oropharyngeal swabs. The median age of the 36 COVID-19 patients was 63 years (interquartile range 55 to 76 years), and 42% (15/36) of these individuals were female. The disease severity of the COVID-19 patients was categorized according to the WHO guideline ‘Clinical Management of COVID-19′ [39]: asymptomatic (no clinical signs of infection), mild (symptomatic patients without evidence of viral pneumonia or hypoxia), moderate (clinical signs of pneumonia, including fever, cough, and dyspnea), severe (clinical signs of pneumonia, plus one of the following: respiratory rate > 30 min^−1^, severe respiratory distress, and peripheral oxygen saturation (SpO_2_) < 90% on room air), critical (one of the following: acute respiratory distress syndrome, sepsis, and septic shock). None of the patients were categorized as asymptomatic, 3 patients were categorized as mild, 8 patients were categorized as moderate, 12 patients were categorized as severe, and 13 were categorized patients as critical. Furthermore, serum samples of 12 healthy donors were included as the control group. These specimens were drawn no later than August 2019 and thus represent immunologically naive samples with respect to SARS-CoV-2 infection. The median age of the 12 healthy and naive donors was 32 years (interquartile range 30 to 53 years), and 67% (8/12) of these individuals were female. Additionally, serum samples of 13 COVID-19 vaccinees, drawn between February 2021 and September 2021, were included in this study. The median age of the 13 vaccinees was 60 years (interquartile range 45 to 67 years), and 54% (7/13) of these individuals were female. All fully vaccinated donors received two shots of the EMA-authorized COVID-19 vaccines, BNT162b2 (Comirnaty, BioNTech, Mainz, Germany) and mRNA-1273 (Spikevax, Moderna Biotech, Cambridge, MA, USA) or AZD1222 (Vaxzevria, AstraZeneca, Cambridge, UK), either in a homologous or heterologous prime-boost scheme. Blood sampling was conducted at least 13 days after the second injection (median 19 days, interquartile range 14 to 33 days) to ensure seroconversion. Blood donors are part of the COVID-19 registry of the LMU Klinikum (CORKUM, WHO trial id DRKS00021225), and the study was approved on 23 March 2020 by the ethics committee (no. 20–245) of the Faculty of Medicine of the LMU (Ethik-Kommission bei der Medizinischen Fakultät der Ludwig-Maximilians-Universität München, Pettenkoferstr. 8a, 80336 München, Germany). Clinical data were obtained from health records, and personal data were anonymized for analyses [29].

## 3. Results

### 3.1. Manufacture of SARS-CoV-2 VLPs and Recipient EVs

Initially, protocols were established to generate suitable cell-free preparations of VLPs and EVs, which were then thoroughly characterized and used in all subsequent experiments. As described previously, VLPs based on SARS-CoV-2, termed S^+^ VLP, were generated in HEK293T cells via the transient expression of the four viral structural proteins S, M, N, E, and CD63~HiBiT, a chimeric reporter protein of human CD63 and the activator of split nanoluciferase HiBiT [29]. S^+^ VLPs (S, M, N, E, CD63∼HiBiT)^+^ were harvested from the cell culture media and further purified if indicated. Recipient EVs were derived from U251MG cells engineered to express ACE2 and CD63~LgBiT, a chimera consisting of CD63 and the major part of the split nanoluciferase reporter protein LgBiT, to tether LgBiT [32] to the inner leaf of the vesicles’ membranes.

### 3.2. Association of SARS-CoV-2 VLPs and ACE2 Vesicles

**Figure 1:**
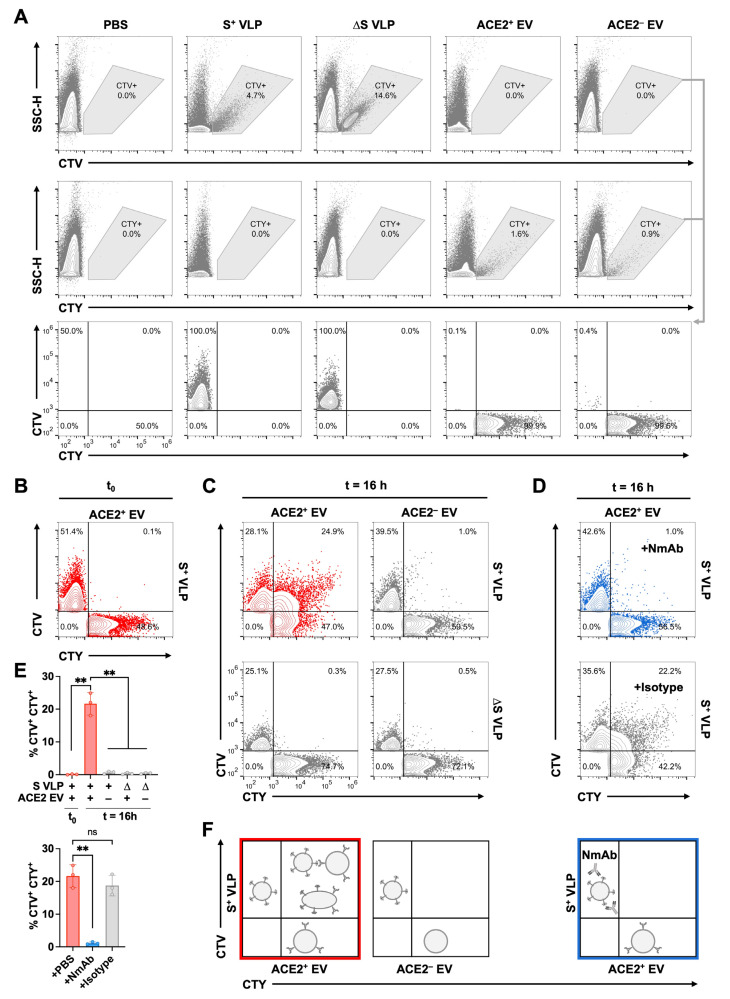
Association of SARS-CoV-2 VLP and ACE2 vesicles. (**A**) Purified SARS-CoV-2 virus-like particles (VLPs) generated by HEK293T cells and U251MG-derived extracellular vesicles (EVs) were stained with CTV or CTY, respectively, and analyzed via flow cytometry. After gating on side scatter height (SSC-H) and CTV or CTY, events in both gates as indicated were merged in the bottom set of panels. Particle-free diluent PBS was used as the control. (**B**,**C**) S^+^ or ∆S VLPs (CTV^+^) were incubated with ACE2^+^ or ACE2^−^ EVs (CTY^+^) at a 1:1 ratio at 37 °C for 0 or 16 h and subjected to flow cytometric analysis. No double-positive CTV^+^ CTY^+^ events could be detected at t_0_ or at t = 16 h in the absence of spike (∆S) or the host receptor (ACE2^−^), but co-association was observed exclusively between S^+^ VLPs and ACE2^+^ EVs. (**D**) The pre-incubation of S^+^ VLPs with the neutralizing monoclonal antibody (NmAb) 35B12 efficiently prevented co-association with ACE2^+^ EVs, while an isotype control IgG mAb did not. (**E**) Experiments from panels (**B**–**D**) were carried out in triplicates, and the percentages of double-positive CTV^+^ CTY^+^ events were summarized. Results from independent *t*-tests are indicated; ns, not significant (*p* > 0.05); ** *p* ≤ 0.01. (**F**) The panels provide a schematic illustration of the experimental situations found in the top row of panels (**C**,**D**) above.

To investigate the co-association of S^+^ VLPs and recipient EVs at the level of single particles and determine their fraction, we used a nanoflow technique as described [29]. VLPs and EVs were stained with ‘CellTraceViolet’ (CTV) and ‘CellTraceYellow’ (CTY), respectively, which exhibit distinct fluorescence after enzymatic ester hydrolysis in the lumen of intact vesicles. To distinguish vesicles from instrument noise, we pre-gated EVs and VLPs on CTV and CTY, respectively, and on side scatter height (SSC-H). The Co-detection of CTV- and CTY-positive events allowed the identification of associated CTV^+^ CTY^+^ particles (Figure 1A). HEK293T-derived S^+^ VLPs or S-negative VLPs, termed ∆S VLPs, were stained with CTV, while U251MG-derived ACE2^+^ EVs or ACE2-negative EVs, termed ACE2^−^ EVs, were stained with CTY. After the adjustment of particle concentrations, individual samples and a vesicle-free diluent control (PBS) were analyzed via flow cytometry, as shown in Figure 1A, while the instrument’s background was successfully excluded. As shown by the diluent control, the four individual samples, S^+^/∆S VLP, and ACE2^+/−^ EV could be separated with little to no spillover of CTV and CTY (Figure 1A).

Next, CTV^+^, S^+^ VLPs and CTY^+^, ACE2^+^ EVs were mixed in a 1:1 stoichiometry and subjected to flow cytometric analyses immediately after mixing (t_0_) and 16 h later (Figure 1B). At t_0_, two single-positive populations were detected, indicating that the vesicles did not spontaneously associate. In contrast, after 16 h of incubation at 37 °C, a third population of CTV^+^ CTY^+^ double-positive particles became obvious (Figure 1C). This population likely originated from co-associated S^+^ VLPs and ACE2^+^ EVs that formed doublets (or multimers) or even underwent membrane fusion (Figure 1F). The remainders of single-positive populations were still detectable, suggesting that not all particles are co-associated probably due to kinetic limitations or missing surface proteins. For instance, S^+^ VLP preparations harbor a fraction of approximately 38% S^+^ particles [29], suggesting that CTV^+^ yet ∆S particles cannot associate with ACE2^+^ EVs. To analyze the underlying specificity of VLP–EV-association, S^+^ or ∆S VLPs were incubated with ACE2^+^ or ACE2^−^ EVs in a panel of four possible combinations (Figure 1C,E). Of these, only S^+^ VLPs with ACE2^+^ EVs yielded a significant fraction of CTV^+^ CTY^+^ double-positive particles after incubation, indicating that co-association is S- and ACE2-mediated and specific. To validate this finding, we pre-incubated S^+^ VLPs with either a SARS-CoV-2-neutralizing monoclonal antibody (NmAb), 35B12, or an isotype control antibody prior to the addition of ACE2^+^ EVs. The NmAb effectively prevented S^+^ VLP association with their target vesicles while the control mAb had no effect (Figure 1D,E). Thus, we show that S^+^ VLPs co-associate with ACE2^+^ EVs in a receptor–ligand-specific manner and in a cell-free environment.

### 3.3. Fusion of SARS-CoV-2 VLPs and ACE2 Vesicles

**Figure 2:**
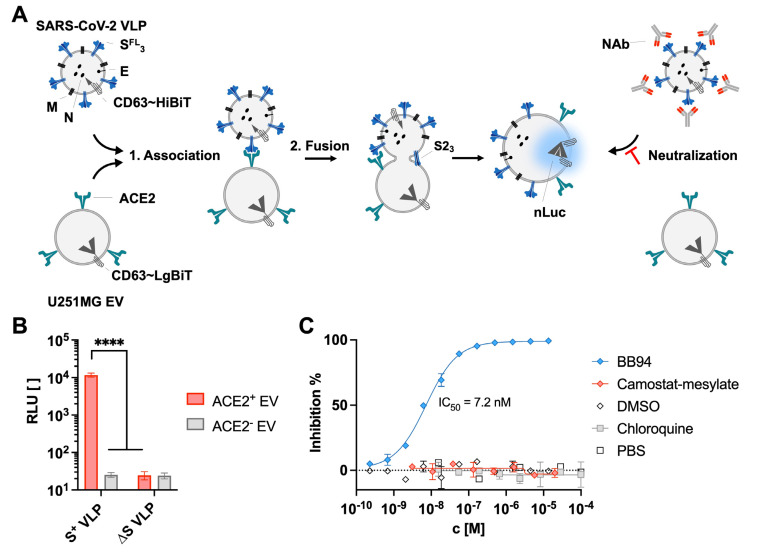
Fusion of SARS-CoV-2 VLP and ACE2 vesicles. (**A**) SARS-CoV-2 VLPs that comprise the viral structural proteins M, N, and E and trimeric, full-length (FL) S protein were engineered to carry CD63~HiBiT, a chimeric protein that anchors the activating peptide of split nanoluciferase (nLuc) inside the lumen. Susceptible EVs derived from U251MG cells and decorated with the host receptor ACE2 were engineered to bear CD63~LgBiT, the complementary entity of the split enzyme. Upon incubation, both particles associate via ACE2 and S, leading to the fusion of membranes and ultimately the reconstitution of functional nLuc. Neutralizing antibodies (NAbs) with specificity for spike inhibit this process and can be quantified accordingly via the reduction in luminescence intensity. (**B**) Preparations of CD63∼HiBiT^+^ and S^+^- or S-deficient (∆S) VLPs were incubated at 37 °C for 4 h with CD63∼LgBiT^+^ and ACE2^+^ or ACE2^−^ EVs and subsequently analyzed for luminescence upon the addition of substrate. High relative light units (RLUs) and thus the fusion of particles were exclusively detected in the presence of viral fusion protein S and host receptor ACE2, confirming the specificity and tropism. Data are based on four independent experiments, and results from independent *t*-tests are indicated; **** *p* ≤ 0.0001. (**C**) To study the mechanism of S^+^ VLPs and ACE2^+^ EVs fusion, both classes of vesicles were incubated with increasing concentrations (c [M]) of protease inhibitors BB94, camostat-mesylate, and chloroquine or control diluents (DMSO and PBS) prior to luminescence analysis. Chloroquine, which prevents endosomal acidification, and camostat-mesylate, a TMPRSS2 inhibitor, did not prevent vesicle fusion while BB94 (batimastat), a broad inhibitor of matrix metalloproteinases (MMPs), effectively blocked fusion. Mean values of three biological replicates are displayed, with error bars indicating standard deviations.

The infection of a host cell is generally initiated by the interaction of a viral glycoprotein with its cellular surface receptor, the uptake of the virion via plasma membrane fusion or fusion with endosomal membranes, and the release of the viral genome followed by virus replication. As reported, S^+^ VLPs are fusogenic, enter permissive target cells like SARS-CoV-2 virions [29], and fuse with ACE2^+^ EV [33]. To validate these findings, we engineered S^+^ VLPs and ACE2^+^ EVs to carry HiBiT and LgBiT, respectively, and they anchored to the vesicle membranes such that the two complementary parts of the enzyme are located intraluminally (Figure 2A).

The fusion of S^+^ VLPs (S, M, N, E, and CD63~HiBiT^+^) and ACE2^+^ EVs (CD63~LgBiT^+^) was traced by measuring enzyme reconstitution with a suitable substrate and the detection of luminescence. Neither VLP nor acceptor EV preparations alone showed detectable background luminescence in the presence of the substrate. However, the incubation of S^+^ VLP with ACE2^+^ EV at 37 °C for 4 h resulted in strong luminescence that is indicative of the fusion events of both classes of vesicles (Figure 2B), which is in line with the results shown in Figure 1C. To better understand the kinetics of this process, S^+^ VLPs were incubated with either ACE2^+^ U251MG cells or ACE2^+^ EVs for various periods of time (Figure S1A,B). After a short lag phase of approximately 5 min, luminescence increased steadily and linearly in both systems within a 6 h observation period. This finding likely reflects the diffusion kinetics of S^+^ VLPs, limiting productive fusion events followed by enzyme reconstitution.

To elucidate the fusion mechanism, ACE2^+^ EVs and S^+^ VLPs were both pre-treated with either chloroquine or camostat-mesylate. While chloroquine deacidifies endosomes and thus inactivates the endosomal, pH-dependent cysteine protease CTSL [40], camostat-mesylate is a serine protease inhibitor that inhibits TMPRSS2 [41]. Both compounds have been shown to block the processing of S and thus the entry of SARS-CoV-2 in certain cell lines in vitro [15,42]. However, neither drug affected VLP–EV-fusion (Figure 2C). The role of the endocytic pathway in a cell-free setting was unlikely, but the result with camostat-mesylate was unexpected because S processing during fusion with ACE2^+^ U251MG cells largely depends on TMPRSS2 [29]. We also tested the impact of BB94 (batimastat), a broad inhibitor of matrix metalloproteinases (MMPs) [43], and observed that it entirely inhibited the fusion of S^+^ VLPs and ACE2^+^ EVs with an IC_50_ of 7.2 nM (Figure 2C). Thus, S processing and activation seem to be catalyzed by a metalloprotease on ACE2^+^ EVs.

**Figure 3:**
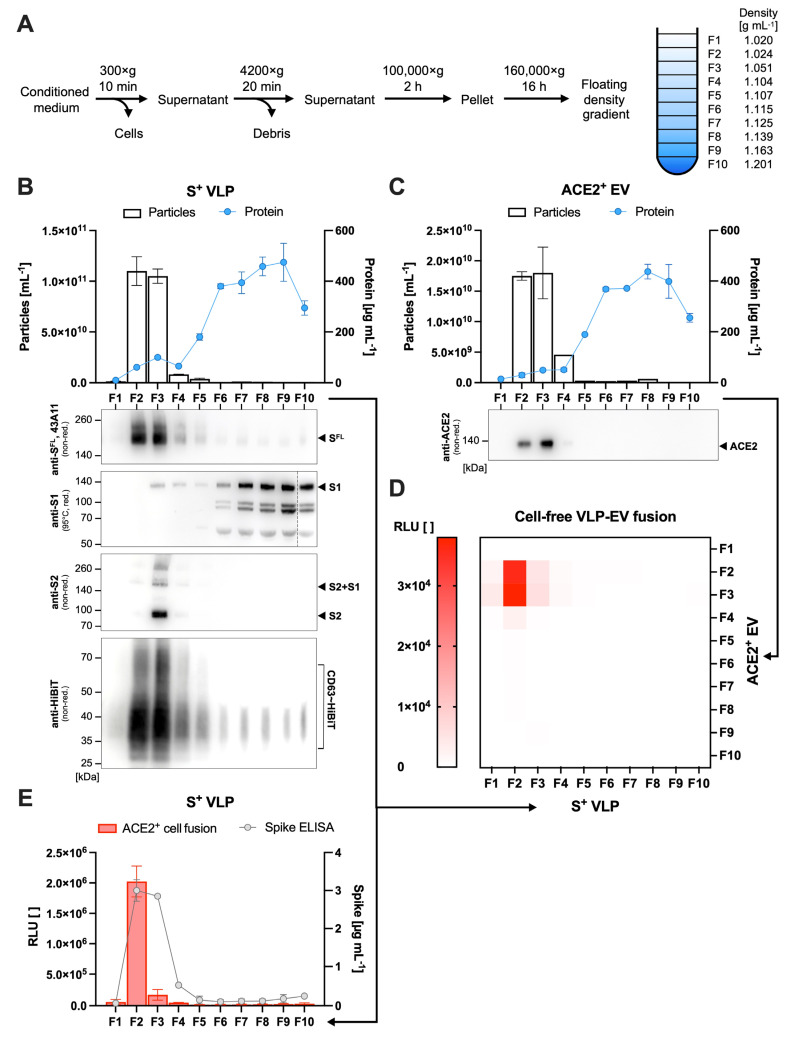
Characterization of purified particles. (**A**) S^+^ VLPs and ACE2^+^ EVs were purified from conditioned cell culture medium by differential centrifugation, sedimentation by ultracentrifugation, and fractionation in a flotation density gradient according to their specific weight. (**B**,**C**) Fractions were examined via BCA and NTA assays for total protein and total particle concentrations, respectively. Additionally, fractions were analyzed by reducing (red.) and non-reducing (non-red.) western blots, using anti-S antibodies specific for full-length (FL) spike, S^FL^ or subunits S1 and S2, and antibodies specific for HiBiT or ACE2. (**D**) Combinations of S^+^ VLPs and ACE2^+^ EV fractions were incubated and analyzed for luminescence, and the resulting relative light units (RLU) were displayed in the heat map. Reconstitution of split nLuc reporter, i.e., VLP–EV fusion, was found to be limited to fraction F2 of S^+^ VLPs and fractions F2 and F3 of ACE2^+^ EVs. (**E**) S^+^ VLP fractions were further analyzed in a specific sandwich ELISA for S^FL^ concentration and the cell-based assay with ACE2^+^, LgBiT^+^ U251MG recipient cells for fusion.

To characterize the fusion of VLPs with EVs in detail, we purified both types of particles by sedimentation and flotation in density gradients according to their specific weight (Figure 3A). Ten fractions F1–F10 of the gradients with increasing densities from top to bottom were analyzed for particle numbers (nanoparticle tracking analysis (NTA)), total protein content, western blot (WB) immunodetection, and an enzyme-linked immunosorbent assay (ELISA) (Figure 3B,C,E). Both classes of particles were almost exclusively found in fractions F2 and F3 (Figure 3B). Because of their lipid membrane composition, the specific weight of VLPs and EVs allows their separation from, e.g., soluble proteins that were found in higher-density fractions F5 to F10. Specific protein analysis by WB revealed ACE2^+^ EVs only in fractions F2 and F3 (Figure 3C). Similarly, the WB analysis of S^+^ VLP preparations with antibodies against the full-length (FL) S protein or HiBiT identified S^FL^ and highly glycosylated CD63∼HiBiT mostly in fractions F2 and F3 (Figure 3B). The data indicated that relevant proteins such as S^FL^, ACE2, and HiBiT copurify and are associated with membranous vesicles.

Next, S^+^ VLPs and ACE2^+^ EVs from individual fractions were combined pairwise in a matrix scheme, incubated at 37 °C for 4 h, and analyzed for luminescence (Figure 3D). As expected, relevant signals indicative of particle fusions were only observed with fractions F2 and F3 of ACE2^+^ EVs. Surprisingly, only fraction F2 of S^+^ VLPs resulted in a strong signal, whereas fraction F3 did not, although both fractions contained equal numbers of particles and were similar with respect to S^FL^ and CD63∼HiBiT content. To confirm this finding, fractions F1-F10 of S^+^ VLPs were re-analyzed in the cell-based assay with LgBiT^+^ and ACE2^+^ U251MG receptor cells (Figure 3E), as described [29]. Similarly, fraction F2 of S^+^ VLPs was highly fusogenic, whereas F3 gave only low signals (Figure 3B,E). WB with anti-S1 and anti-S2 antibodies revealed that F2 was completely devoid of the cleaved S1 subunit, whereas cleaved S2 and complexes of co-associated S1/S2 were identified in F3 (Figure 3B). Of note, most of the free S1 protein was detected in fractions from F6 onwards with high total protein contents.

We hypothesized that cleavage at S1/S2 is mandatory for VLP–EV fusion as it is for viral entry into cells [44]. Thus, a mutant with a deleted furin cleavage site, S^∆Furin^, was constructed, which contains three amino acid substitutions of arginine to delete the protease site RRAR at positions 682–685 of spike [45]. S^+^ or S^∆Furin^ VLPs were generated, compared for particle concentration by NTA, and analyzed for fusion with cells or ACE2^+^ EV. In concordance with the literature on the role of furin for SARS-CoV-2 [46,47], S1/S2 cleavage supported fusion with ACE2^+^ U251MG cells, but they were found to be mandatory for fusion with EVs derived from these cells (Figure S2).

Taken together, experiments with purified and thoroughly characterized S^+^ VLP and ACE2^+^ EV stocks confirmed their fusogenic characteristics. Fusion was highly specific as it depended on ACE2 and processed spike proteolytically cleaved by an MMP. These findings fulfill important requirements for the development of a cell-free virus neutralization test to quantitate virus-neutralizing antibodies.

### 3.4. Cell-Free SARS-CoV-2 Neutralization Test

Virus-neutralizing antibodies (NAbs) mediate protection from SARS-CoV-2 infection as they block virus entry into host cells. The majority of S-specific Nabs prevent attachment of S to ACE2 by occupying and blocking the receptor binding motif (RBM) of S1 [48,49] or locking the RBD in an inaccessible closed structural state [50]. Other antibodies neutralize without disrupting the interaction with ACE2 [51] but by inhibiting the proteolytic processing of S or by interfering with the conformational rearrangement of S2, as shown for SARS-CoV or MERS-CoV [52,53]. As NAbs also interfere with the entry of S^+^ VLP into susceptible target cells, we recently demonstrated their reliable quantification in a VNT using VLPs (VLPNT) instead of infectious virus stocks [29]. Similarly, we wanted to assess whether a completely cell-free assay based solely on S^+^ VLP and ACE2^+^ EV can be developed (Figure 2A). To that end, defined amounts of S^+^ VLPs were pre-incubated with serial dilutions of serum samples for 30 min and then mixed with ACE2^+^ EVs. After 4 h, the substrate was added, and luminescence was quantified. The mean luminescence of S^+^ VLPs in the absence of serum and the background luminescence obtained with ∆S VLP were normalized to 0% and 100% neutralization, respectively. The titers were determined by extrapolating sigmoidal curve values that correspond to 50% absolute signal reduction after background correction. This value was termed VLPN_50_ and is considered equivalent to the VNT_50_ and PRNT_50_ of cVNTs and plaque reduction neutralization tests (PRNTs).

**Figure 4:**
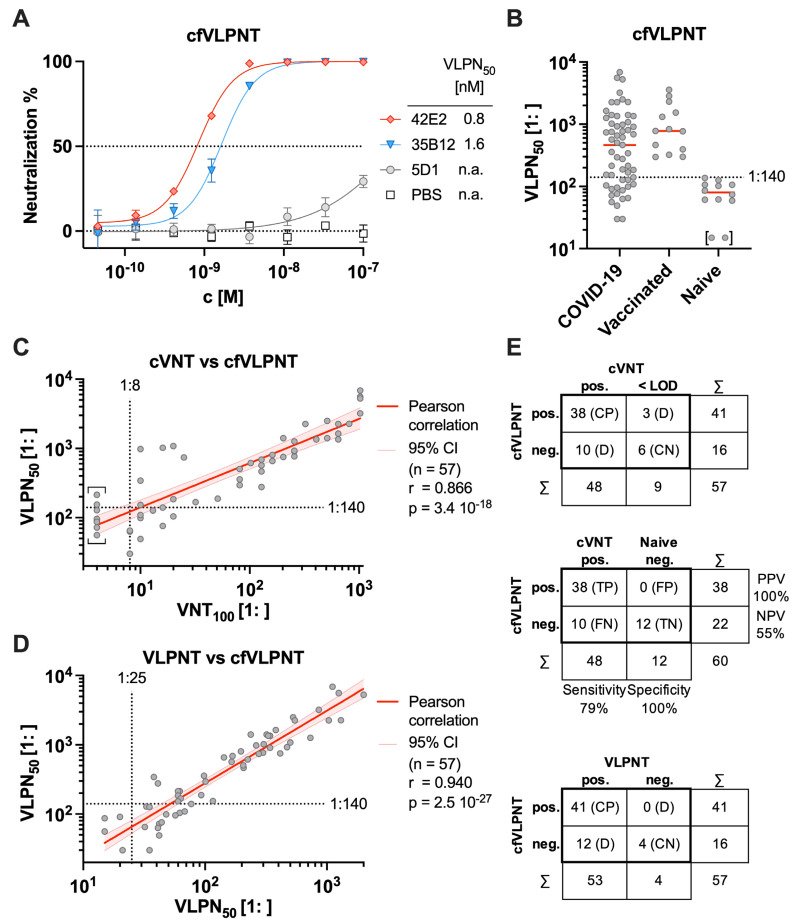
Validation of the cell-free SARS-CoV-2 virus neutralization test. (**A**) Neutralizing (42E2, 35B12) and non-neutralizing (5D1) anti-S specific monoclonal antibodies were analyzed in the cell-free VLP neutralization test (cfVLPNT) for the neutralization of S^+^ VLP fusion with ACE2^+^ EVs. Mean values of three biological replicates are displayed, and error bars indicate standard deviations. The 50% neutralization values, VLPN_50_, are listed or indicated as not applicable (n.a.). (**B**) Neutralization titers of 57 COVID-19 patients, 13 COVID-19 vaccinee, and 12 control serum specimens from healthy and immunologically naive donors tested in the cfVLPNT with S^+^ VLPs, are shown. Median values are indicated in red. Samples above the dotted line (1:≥140) scored positive according to the criteria applied. Samples in brackets indicate sera with a VLPN_50_ below the limit of detection (LOD). (**C**) VLPN_50_ titers from the cfVLPNT are plotted along VNT_100_ titers obtained in a conventional virus neutralization test (cVNT) with infectious SARS-CoV-2. Pearson correlation data (coefficient *r*, confidence interval CI, sample size *n*, and *p*-value) of 57 sera from COVID-19 patients are shown. Results left of the dotted vertical line denote sera, which scored below the LOD (1:<8) in the cVNT; these VNT_100_ values were defined as 1:4 and indicated in square brackets. (**D**) VLPN_50_ titers from the cell-free (vertical axis) and the cell-based (horizontal axis) VLPNT are plotted. As in panel (**C**), Pearson correlation data of 57 sera from COVID-19 patients are shown. Results left of the dotted vertical line denote sera, which scored negative (1:<25) in the VLPNT. (**E**) In the top table, the results of 57 COVID-19 samples tested in the cfVLPNT and a cVNT are compared. In the middle table, the sensitivity, specificity, positive predictive value (PPV), and negative predictive value (NPV) of the cfVLPNT were calculated based on 48 COVID-19 serum samples with neutralizing capacity according to the cVNT and 12 controls of immunologically naive donors. In the bottom table, the results of 57 COVID-19 samples tested in the cfVLPNT and the cell-based VLPNT are compared. Test results are indicated: pos., positive; neg., negative; <LOD, below the limit of detection; CP, concordant positive; CN, concordant negative; D, discrepant; TP, true positive; FN, false negative; FP, false positive; TN, true negative.

To demonstrate the proof of principle of a cell-free VLP neutralization test (cfVLPNT), we tested three in-house generated anti-S mAbs, two of which block viral infection (Figure 4A). NmAbs 42E2 and 35B12 showed strong neutralization with VLPN_50_ at 0.8 and 1.6 nM, respectively, while mAb 5D1 was not functional as expected.

Next, we used human serum samples to demonstrate the applicability of the cfVLPNT as a serological assay and diagnostic test. Sera from 12 healthy and immunologically naive donors obtained before mid-2019 or earlier and sera from 13 COVID-19 vaccinees after prime-boost immunization in 2021 were tested. Of the 12 presumably SARS-CoV-2 seronegative samples, 10 reached levels of 50% neutralization, with some showing VLPN_50_ titers as high as 1:137, indicating the non-specific interference of serum components with the assay reagents. Therefore, a lower cutoff of 1:≥140 was defined to ensure specificity for in vitro SARS-CoV-2-neutralizing antibodies. In contrast, titers from the vaccinated individuals consistently revealed VLPN_50_ titers between 1:297 and 1:3274. Thus, 13 sera out of 13 were found to contain SARS-CoV-2 neutralizing antibodies by our criteria (Figure 4B). Next, 57 well-documented clinical serum samples from 36 COVID-19 patients with acute or convalescent SARS-CoV-2 infection were tested under double-blinded, randomized conditions in the cfVLPNT. Their VLPN_50_ titers ranged from 1:30 to 1:6863 and 41 of 57 specimens were found to neutralize according to the cutoff criteria (Figure 4B). Yet, very weakly neutralizing samples could not unambiguously be discriminated from non-neutralizing serum samples.

### 3.5. Comparison of Virus Neutralization Tests

To validate the results from our assay, 57 COVID-19 sera were tested in a cVNT with replication-competent SARS-CoV-2 [38]. Titers were determined based on the 100% reduction in CPE (VNT_100_), and values ranged from 1:8 to 1:>1024. Thereof, 48 samples were found to be neutralizing with a VNT_100_ of 1:≥8, while the 9 remaining sera performed below the limit of detection (LOD) and were denoted as 1:4. To compare the cfVLPNT with the cVNT directly, VLPN_50_ titers of the COVID-19 samples were correlated with the respective VNT_100_ titers (Figure 4C). The Pearson coefficient of log_10_-transformed data (*n* = 57) revealed a significant positive correlation (*r* = 0.866 and *p* = 3.4 × 10^−18^), indicating the high accuracy of the novel test. Analyses of the 57 COVID-19 samples in both tests found 38 sera to be concordant positive (CP), 6 to be concordant negative (CN), and 13 to be discrepant (Figure 4E, upper table). The latter is likely attributable to experimental differences between the two tests [6] as the cVNT is a multi-cycle assay that requires 100% reduction in CPE (VNT_100_) while the cfVLPNT is a single-cycle assay and scores at 50% reduction in S^+^ VLP fusion (VLPN_50_). Furthermore, mechanistic differences could cause inconsistencies as the cfVLPNT uses a specific one-step fusion mechanism between S^+^ VLP and ACE2^+^ EV while the cVNT is characterized by several infection rounds of cultivated cells. A calculation of the sensitivity and specificity of the cfVLPNT was based on clearly attributable positive (neutralizing) and negative (non-neutralizing) specimens, using the 48 COVID-19 samples that had scored above the LOD in the cVNT along with the 12 sera from immunologically naive donors (Figure 4E, middle table). Of 48 positive samples, the cfVLPNT determined 38 to be true positive (TP) while all 12 negative sera were considered true negative (TN) with the applied cutoff. Therefore, the sensitivity of the cfVLPNT was calculated to be 79%, while its specificity was 100%. Based on the analyzed dataset (*n* = 60), the positive and negative predictive values (PPV resp. NPV) of the test were 100% and 55%, respectively.

Additionally, results from the cfVLPNT were compared with those from the cell-based VLPNT [29]. Titers in the VLPNT ranged from 1:15 to 1:2000 for the COVID-19 cohort, and 53 out of 57 samples were found to exhibit neutralizing activities with VLPN_50_ values of 1:≥25, while the 4 remaining sera were classified to be negative. To compare the cfVLPNT with the VLPNT directly, VLPN_50_ titers of the 57 COVID-19 samples from both tests were correlated (Figure 4D). The Pearson coefficient of log_10_-transformed data (*n* = 57) revealed a significant and strong positive correlation (*r* = 0.940 and *p* = 2.5 × 10^−27^). The direct comparison of the samples in both tests found 41 sera to be CP and 4 to be CN, whereas 12 sera were found to be discrepant (Figure 4E, bottom table). In particular, the high cutoff of 1:≥140 caused some of the COVID-19 samples to appear as negative in the cfVLPNT, although they do contain NAbs according to the two other tests. Overall, results from the cfVLPNT correlated even better with those from the VLPNT and supported the cell-free assay as a clearly reliable diagnostic method.

### 3.6. Adaptation to SARS-CoV-2 Variants

We also adapted the cfVLPNT with B.1 (Wuhan-2019, D614G) S^+^ VLP to the SARS-CoV-2 variants of concern (VOC): B.1.617.2 (Delta) and B.1.1.529 BA.1 (Omicron). Therefore, the respective particles were generated [29]; pre-incubated with the pan-variant neutralizing mAb 35B12, isotype control antibody, or buffer; and mixed with ACE2^+^ EV (Figure S3A). The resulting luminescence data confirmed successful fusion and neutralization with all VOCs. Subsequently, 35B12 was titrated with the S^+^ VLP of respective variants to estimate the coefficient of variation (CV) of the cfVLPNT between the mean values of individual VOCs (Figure S3B). The resulting VLPN_50_ values ranged from 0.45 to 0.65 nM for B.1, B.1.617.2, or B.1.1.529 BA.1. Thus, an inter-variant CV of 0.18 was calculated, confirming consistency between variants and the successful adaptation of the diagnostic test to other VOCs. Noteworthily, for inter-assay repeatability, a CV of 0.20 was determined with B.1 S^+^ VLPs and 35B12 mAb, but further tests according to Clinical Laboratory Standards Institute (CLSI) guidelines are necessary [54].

### 3.7. Adaptation to EBV

Next, we investigated whether a fusion of VLPs and EVs can also be observed with enveloped viruses other than SARS-CoV-2. To that end, we adapted our methodology to EBV. Vaccine candidates against this human pathogen are currently in development, but simple and reliable tests for the quantitation of neutralizing antibodies do not exist. To generate VLPs from EBV, termed EB-VLPs, a HEK293-based cell line that harbors copies of a recombinant EBV genome with several genetic deletions was engineered such that the cells do not release infectious virions [55,56,57] but express CD63~HiBiT constitutively. The cells also express a chimeric EBV full-length BZLF1 protein, which can be activated by tamoxifen to induce EB-VLP production [37,58]. Upon induction, the cells release CD63~HiBiT-decorated EB-VLPs in high numbers, which were purified via density gradient ultracentrifugation as described above. In parallel, recipient EVs were harvested from a derivative of the Daudi cell line, a B cell, and a permissive recipient of EBV, which was engineered to constitutively express CD63~LgBiT.

**Figure 5:**
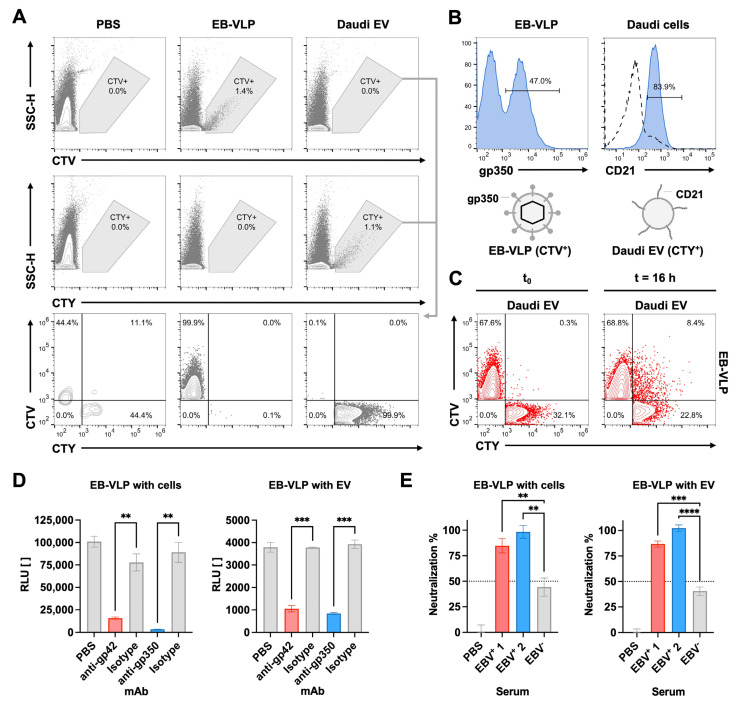
Fusion and neutralization of EB-VLP with vesicles and cells. (**A**) Purified samples of Epstein–Barr virus-like particles (EB-VLPs) and Daudi-cell-derived EVs were stained with CTV or CTY, respectively, and analyzed via flow cytometry. After gating on side scatter height (SSC-H) and CTV or CTY events, both gates were merged, as shown in the bottom row of panels. Particle-free PBS was used as a control. (**B**) EB-VLPs (CTV^+^) and Daudi cells were stained with glycoprotein gp350 and a CD21-specific antibody, respectively, and analyzed via flow cytometry. (**C**) EB-VLPs (CTV^+^) were incubated with Daudi-cell-derived EVs (CTY^+^) at 1:1 stoichiometry at 37 °C for 0 or 16 h and subjected to flow cytometric analyses. No double-positive CTV^+^ CTY^+^ events could be detected at t_0_, and the co-association of the two classes of vesicles was found after 16 h. (**D**) EB-VLPs (CD63~HiBiT^+^) were incubated with CD63~LgBiT^+^ Daudi cells or EVs at 37 °C for 4 h prior to the addition of substrate and luminescence analyses. Pre-incubation of EB-VLPs with 10 µg mL^−1^ monoclonal antibodies (mAb) specific for EBV antigens gp42 or gp350 effectively prevented fusion with Daudi cells and Daudi-cell-derived EVs while IgG isotype controls did not. Results from independent *t*-tests with biological triplicates are indicated; ** *p* ≤ 0.01; *** *p* ≤ 0.001. (**E**) EB-VLPs were pre-incubated with 1:50 diluted human sera from two EBV-seropositive and one EBV-negative donor one hour prior to incubation with Daudi cells or EVs. Signals were normalized to diluent PBS control and analyzed for neutralization. Results from independent *t*-tests based on triplicates are indicated; ** *p* ≤ 0.01; *** *p* ≤ 0.001; **** *p* ≤ 0.0001.

First, we analyzed EB-VLPs and Daudi EVs for their co-association after staining with CTV and CTY, respectively, followed by nanoflow cytometry. Similarly, the single preparations were fluorescent only in their corresponding color (Figure 5A). EB-VLPs were also analyzed for the major glycoprotein of EBV, gp350, which mediates attachment to human CD21 on B cells. The staining of CTV^+^ EB-VLP with the fluorescently labelled anti-gp350 mAb 6G4 revealed about 47% positive particles within the preparations used (Figure 5B). Next, CTV^+^, EB-VLPs, and CTY^+^ Daudi EVs were quantified via NTA, mixed at 1:1 stoichiometry, and subjected to flow cytometric analysis immediately at t_0_, or after 16 h of incubation at 37 °C (Figure 5C). While no double-positive events could be detected at t_0_, a population of CTV^+^ CTY^+^ double-positive particles was found after 16 h, which is indicative of either the co-association or membrane fusion of EB-VLPs and Daudi EVs.

Subsequent experiments in Figure 5D demonstrated that CD63~HiBiT^+^ EB-VLPs are fully functional, as they were efficiently taken up by CD63~LgBiT^+^ Daudi cells. Entry into these cells was specifically mediated by EBV’s glycoproteins because the pre-incubation of EB-VLPs with NmAbs against gp42 and gp350 effectively prevented uptake by probably interfering with attachment. To test the cell-free fusion of EB-VLPs with Daudi EVs, both types of vesicles were mixed and incubated for 4 h at 37 °C. Distinct luminescence confirmed membrane fusion and nanoluciferase reconstitution (Figure 5D). As expected, the pre-incubation of both preparations with NmAbs against gp42 and gp350 suppressed fusion, while isotype control antibodies did not. Therefore, EB-VLPs are not only taken up by susceptible cells but also fused with suitable EVs specifically mediated by EBV glycoproteins.

To demonstrate the proof-of-principle of a cell-free EBV neutralization test, human sera from two EBV-seropositive and one EBV-seronegative donor were diluted at 1:50 and pre-incubated with EB-VLPs, combined with Daudi EVs, and incubated for 4 h at 37 °C. Using controls, luminescence was normalized to 0% neutralization in the absence of serum and to 100% with EB-VLPs fully blocked with NmAb. While the control serum of the EBV-negative donor only showed weak non-specific effects, sera from the two EBV-positive donors displayed strong neutralization (Figure 5E). To verify these findings, the sera were similarly tested in the EBV neutralization test with Daudi cells as targets instead of acceptor EVs, which confirmed the results unambiguously (Figure 5E). Therefore, a semi-quantitative analysis of EBV-neutralizing serum antibodies with EB-VLPs and recipient EVs obtained from Daudi cells was realized in a cell-free setting.

## 4. Discussion

We recently reported that S^+^ VLPs are morphological mimics of SARS-CoV-2 and faithfully recapitulate the earliest steps of viral infection. S^+^ VLP attaches to receptors on susceptible cells and ‘infect’ them via endocytosis or direct fusion with the plasma membrane [29]. Based on our findings and reports of VLP–EV fusion [33], we investigated whether we could apply this principle to a VNT. The finding that VLPs fuse with EVs in a specific process might have conceivable relevance in vivo. El-Shennawy et al. have shown recently that ACE2^+^ EVs can act as decoys that compete with the binding of SARS-CoV-2 virions to recipient cells and consequently might protect transgenic mice from infection [59].

We confirm that S^+^ VLP and ACE2^+^ EV co-associate and fuse efficiently, mediated by the viral glycoprotein S and its interaction with the cognate host receptor (Figure 1 and Figure 2B). To test fusion, we used the split nanoluciferase protein reporter and tethered both entities to the inside of vesicles via CD63 [32], a tetraspanin protein prevalent in the membranes of extracellular vesicles. Next, we investigated the underlying mechanisms of VLP–EV fusion. Indistinguishable from the infection of target cells, fusion with EVs was mediated exclusively by the viral fusion protein spike, and the VLPs showed a clear tropism for receptor ACE2 (Figure 2B). In contrast to fusion with U251MG cells, the fusion of S^+^ VLPs with ACE2^+^ EVs was independent of the accessory protease TMPRSS2 (Figure 2C) but was completely inhibited by broad MMP inhibitor BB94, suggesting that the spike is cleaved by a yet unspecified protease on the surface of recipient EVs. Possible candidates could be ADAM17 or other MMPs, which have recently been proposed to mediate processing at the S2′ site during SARS-CoV-2 entry at the plasma membrane [60,61]. Analyses of purified S^+^ VLPs found them to be largely proteolytically primed at the S1/S2 site, which likely occurred during egress [29]. Via density gradient centrifugation, we identified two distinct populations of S^+^ VLPs of different specific weights, spike protein composition, and fusogenicity (Figure 3). Unexpectedly, the fraction of S^+^ VLPs with a primed spike protein did not readily support fusion with ACE2^+^ U251MG cells or the EVs derived from them. This observation is in contrast with reports that found furin cleavage to be dispensable for SARS-CoV-2 infection [46]. Subsequent experiments with VLPs and an RRAR deletion variant of spike, S^∆Furin^, were unsuccessful because fusion with ACE2^+^ EVs was blocked. It thus appears that the fusion mechanism of S^+^ VLPs with ACE2^+^ EV also differs slightly from entry into susceptible cells with respect to the usage of TMPRSS2 (or an alternative metalloprotease).

The SARS-CoV-2 pandemic made clear that vaccine development and immune monitoring require reliable in vitro diagnostics. Serum titers of NAbs were found to be a good correlate of protection from COVID-19 and are consequently used to assess the immune protection of vaccinees, patients, convalescents, and people at risk [3,4,5]. Conventional methods for the quantitation of NAbs depend on replication-competent viruses and thus have to be conducted in BSL-3 containment laboratories, and they mostly rely on in vitro CPEs and the formation of plaques in infected cell monolayers. The plaques, which take several days to evolve, need to be counted microscopically by trained personnel, which is a labor-intensive technique that is difficult to normalize and standardize between laboratories [6]. Instead, a microneutralization assay (MNA) can be used, which relies on the identification of infected cell clusters after immunostaining. While this type of assay does not depend on a microscopically visible CPE, it requires an infectious virus, numerous steps for completion, and takes days [7,62,63]. Alternatively, pVNTs use replication-deficient viral vectors with a reporter gene and the viral fusion protein of concern. For retroviral scaffolds, the readout requires infection, reverse transcription, chromosomal proviral DNA integration, de novo transcription, and translation. Thus, the assay requires two to three days and a BSL-2 laboratory but tests the spike as the only SARS-CoV-2-derived component. Although pVNTs are widely used, the assembly, morphogenesis, structure, and composition of such particles can significantly differ from the original virus [6]. Another simplistic approach are surrogate tests that quantify the interaction between two protein domains of the host receptor and the interacting viral glycoprotein. These tests neither require living cells nor infectious viruses but measure antibodies that interfere with protein–protein interactions only. For SARS-CoV-2, such anti-RBD antibody levels display rather weak correlations to titers from cVNTs [7,8,9].

In this context, the fusion of S^+^ VLP and ACE2^+^ EV might offer a new principle for measuring NAb titers in serum, as shown by our cfVLPNT and recently also reported by Kicmal et al. [64]. The authors describe an assay that is similar to ours and includes coronaviruses other than SARS-CoV-2. In contrast to their approach, our S^+^ VLPs do not depend on the tryptic activation of S but are rather activated by an inherent MMP on ACE2^+^ acceptor EVs. Furthermore, our study also demonstrates the practicality of the approach with the analysis of an extensive set of 82 human serum samples of COVID-19 patients, vaccinees, and immunologically naive donors. Seronegative specimens showed weak neutralization, probably due to interfering matrix effects from serum components or the cross-reactivity of antibodies against endemic coronaviruses. Consequently, very low titers were indistinguishable from negative samples, thus requiring a high cutoff value to ensure test specificity. Importantly, the NAb titer VLPN_50_ of the tested COVID-19 samples were compared to their results in a CPE-based cVNT with infectious virus (Figure 4C,E) [38]. Results from both tests showed strong positive correlations and allowed for the calculation of preliminary test parameters. While full specificity and excellent PPV were achieved, lower values for sensitivity and NPV had to be accepted. Despite its current limitations, the cfVLPNT could be verified as a reliable and accurate quantitative novel VNT for SARS-CoV-2 and its VOCs, as titers of the COVID-19 samples from the cfVLPNT correlated well with data obtained from our established, cell-based VLPNT (Figure 4D,E) [29]. Both assays follow simple and quick protocols that require only standard laboratory equipment. The cfVLPNT is even easier because it is independent of living cells and cell culture facilities (Figure S4), and it can be performed quickly and needs little work compared to a cVNT or pVNT. The S^+^ VLP-based tests are superior compared to those with pseudotyped particles as they faithfully reflect the original virus. Nevertheless, the cfVLPNT could benefit from further improvements that reduce non-specific serum effects in order to clearly distinguish them from low levels of neutralizing serum antibodies and increase the overall test’s sensitivity.

We also demonstrate VLP–EV co-association and fusion with another enveloped virus, EBV, which differs from SARS-CoV-2 in many aspects. We successfully used EB-VLPs to quantitate virus-neutralizing antibodies in a cell-free and cell-based VLPNT. NmAbs directed against EBV gp42 or gp350 and serum samples from EBV-seropositive donors specifically neutralized EB-VLP in both assays, confirming the potential of this technology.

In summary, we show that VLPs co-associate and fuse with permissive EVs in vitro and demonstrate that this process can be exploited as a new principle for diagnostic VNTs. Speculating whether the fusion of viruses with EVs also occurs in infected individuals in vivo remains tempting.

## Data Availability

All data are available in the main text or Supplementary Materials.

## Supplementary Materials

The following supporting information can be downloaded at https://www.mdpi.com/article/10.3390/biomedicines11112892/s1. Figure S1: Fusion kinetics of SARS-CoV-2 VLP; Figure S2: Analysis of cleavage resistant Spike mutant S^ΔFurin^; Figure S3: Experiments with SARS-CoV-2 Spike variants; Figure S4: Comparison of cell-based and cell-free VLP neutralization tests; Table S1: Individual data of analyzed human serum samples.

## Abbreviations

%	Percent
°C	Degree Celsius
×g	Gravitational force equivalent
ACE2	Angiotensin-converting enzyme 2
BSL	Biosafety level
c	Concentration
cfVLPNT	Cell-free VLP neutralization test
CN	Concordant negative
conc.	Concentrated
COVID-19	Coronavirus disease 2019
CP	Concordant positive
CPE	Cytopathic effect
CTSL	Cathepsin L
CTV	CellTraceViolet (Thermo Fisher Scientific)
CTY	CellTraceYellow (Thermo Fisher Scientific)
CV	Coefficient of variation
cVNT	Conventional virus neutralization test
D	Discrepant
Da	Dalton
dd	Double distilled
DMEM	Dulbecco’s Modified Eagle’s Medium
DMSO	Dimethyl sulfoxide
DNA	Deoxyribonucleic acid
E	Envelope protein
e.g.	*exempli gratia*
EB-VLP	Epstein-Barr virus-like particle
EBV	Epstein-Barr virus
ELISA	Enzyme-linked immunosorbent assay
EV	Extracellular vesicle
FBS	Fetal bovine serum
FL	Full length
FN	False negative
FP	False positive
FSC	Forward scatter
g	Gram
GMO	Genetically modified organism
GMT	Geometric mean titer
h	Human
HIV	Human immunodeficiency viruses
HRP	Horseradish peroxidase
IC_50_	Half maximal inhibitory concentration
IgG	Immunoglobulin G
IU	International units
k	kilo
kb	kilo base pair
L	Liter
LOD	Limit of detection
log_10_	Decadic logarithm
Luc	Luciferase
M	Membrane protein
m	Meter
m	milli
M	mol L^−1^
mAb	Monoclonal antibody
MERS-CoV	Middle East respiratory syndrome Coronavirus
min	Minutes
MLV	Murine leukemia virus
MMP	Matrix metalloproteinase
MNA	Microneutralization assay
mol	Mole
mRNA	Messenger RNA
N	Nucleoprotein
n	nano
*n*	Sample number
n.a.	Not applicable
na	Not available
NAb	Neutralizing antibody
NIBSC	UK National Institute for Biological Standards and Control
nLuc	nano-Luciferase
NM	*N*-myristoly
NmAb	Neutralizing monoclonal antibody
non-red.	Non-reducing
NPV	Negative predictive value
ns	Not significant
NTA	Nanoparticle tracking analysis
*p*	*p*-value
PAGE	Polyacrylamide gel electrophoresis
PBS	Phosphate buffered saline
PCR	Polymerase chain reaction
Pen	Penicillin
PFU	Plaque-forming units
PPV	Positive predictive value
PRNT	Plaque reduction neutralization test
PRNT_50_	50% plaque reduction neutralization titer
pVNT	Pseudotyped virus neutralization test
*r*	Pearson correlation coefficient
RBD	Receptor binding domain
RBM	Receptor binding motif
red.	Reducing
RLU	Relative light units
RNA	Ribonucleic acid
RT	Room temperature
RT-qPCR	Reverse transcription quantitative real-time PCR
S	Spike protein
SARS-CoV	Severe acute respiratory syndrome Coronavirus
SARS-CoV-2	Severe acute respiratory syndrome Coronavirus 2
SD	Standard deviation
SDS	Sodium dodecyl sulfate
SpO2	Peripheral oxygen saturation
SSC-H	Side scatter height
Strep	Streptomycin
sVNT	Surrogate virus neutralization tests
TBS	Tris-buffered saline
TBST	Tris-buffered saline with 0.1% Tween-20
TMPRSS2	Transmembrane protease serine 2
TN	True negative
TP	True positive
Tris	Tris(hydroxymethyl)aminomethane
V	Volt
VLP	Virus-like-particle
VLPN_50_	50% VLP neutralization titer
VLPNT	VLP neutralization test
VNT	Virus neutralization tests
VNT_100_	100% virus neutralization titer
VNT_50_	50% virus neutralization titer
VOC	Variant of concern
VSV	Vesicular stomatitis virus
w/v	Weight per volume
WB	Western-blot
WHO	World Health Organization
µ	micro
