# Supplementary material for: SARS-CoV-2 and Epstein–Barr Virus-like Particles Associate and Fuse with Extracellular Vesicles in Virus Neutralization Tests
Source: Biomedicines. 2023 Oct 25;11(11):2892. doi: 10.3390/biomedicines11112892 (PMC10669694; doi:10.3390/biomedicines11112892)
Supplement: Supplementary file 1 [file biomedicines-11-02892-s001.zip › biomedicines-2672928-supplementary.pdf]

SARS-CoV-2 and Epstein-Barr virus-like particles associate and fuse with extracellular vesicles in virus neutralization tests

Supporting Information

Authors

Johannes Roessler[1,2,3], Dagmar Pich[2,3], Verena Krähling[4,5], Stephan Becker[4,5], Oliver T. Keppler[3,6,7], Reinhard Zeidler[1,3,9] and Wolfgang Hammerschmidt[2,3*]

Affiliations

[1]Department of Otorhinolaryngology, University Hospital, Ludwig-Maximilians-Universität (LMU) München, Munich, Germany

[2]Research Unit Gene Vectors, Helmholtz Zentrum München, German Research Center for Environmental Health, Munich, Germany

[3]German Centre for Infection Research (DZIF), Partner site Munich, Germany

[4]Institute of Virology, Faculty of Medicine, Philipps University Marburg, Germany

[5]German Centre for Infection Research (DZIF), Partner site Giessen-Marburg-Langen, Marburg, Germany

[6]COVID-19 Registry of the LMU Munich (CORKUM), LMU University Hospital, Munich, Germany

[7]Max von Pettenkofer Institute and Gene Center, Virology, National Reference Center for Retroviruses, Faculty of Medicine, LMU, Munich, Germany

[9]Institute of Structural Biology, Helmholtz Munich, Germany

***Corresponding author**
Wolfgang Hammerschmidt
Feodor-Lynen-Str. 21
D-81377 München
Germany
Phone: +49-89-3187-1506
Fax: +49-89-3187-4225
E-mail: wolfgang.hammerschmidt@helmholtz-munich.de

List of Supplementary Materials

Supplementary Fig S1 to S4

Supplementary Table S1

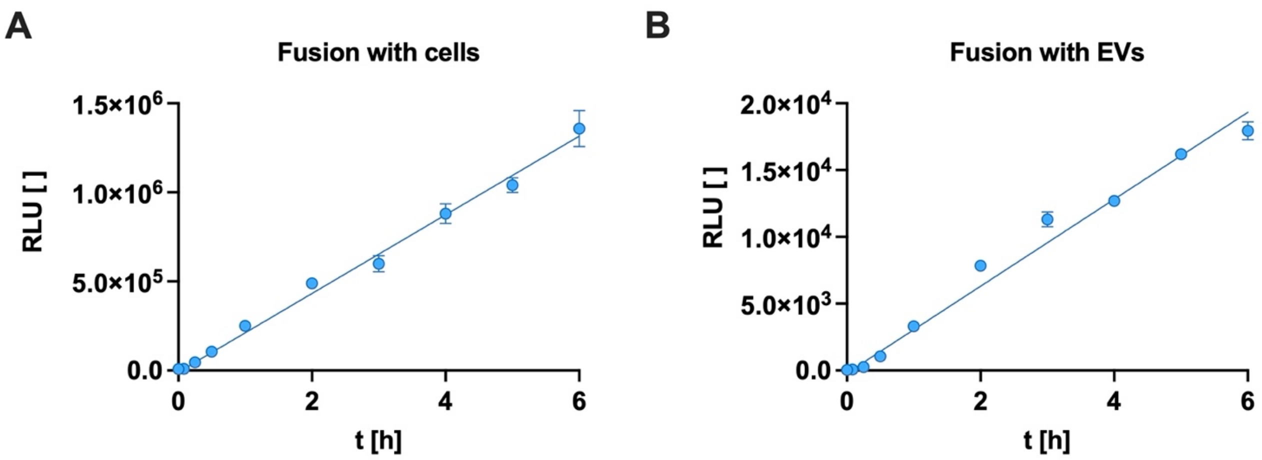

Supplementary Figure S1. Fusion kinetics of SARS-CoV-2 VLP.

(A, B) S^+ VLPs were incubated with $ACE2^+$ U251MG cells (A) or $ACE2^+$ EVs (B) derived from these cells at 37°C for up to 6 h prior to substrate addition and luminescence analysis. The results are depicted as relative light units (RLU). The increase in luminescence after a short lag phase is extrapolated by linear regression.

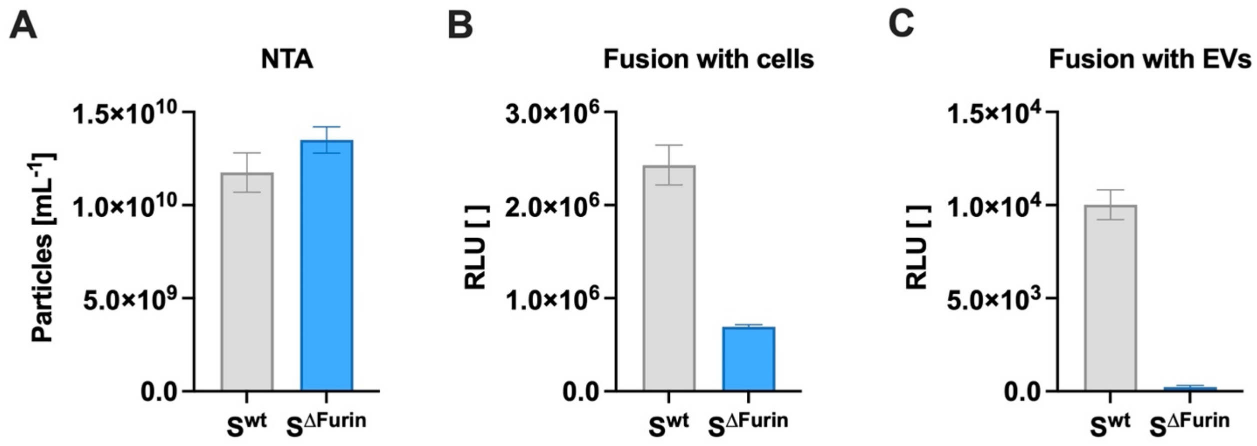

Supplementary Figure S2. Analysis of cleavage resistant Spike mutant $S^{\Delta Furin}$.

(A) S^+ VLPs that either comprise wild-type (wt) SARS-CoV-2 spike or a S1/S2 cleavage deficient mutant with a deleted recognition motif of furin and furin-like proteases, $S^{\Delta Furin}$ were generated in HEK293T cells and analyzed by NTA for physical particle concentration.

(B, C) S^+ VLP (CD63~HiBiT$^+$) of S^{wt} and $S^{\Delta Furin}$ were incubated with ACE2$^+$ U251MG cells (NM~LgBiT$^+$) in panel B or U251MG derived ACE2$^+$ EV (CD63~LgBiT$^+$) in panel C at 37°C for 4 h prior to substrate addition and luminescence analysis. The results are depicted as relative light units (RLU). High signals indicate successful fusion with both target cells and acceptor EVs.

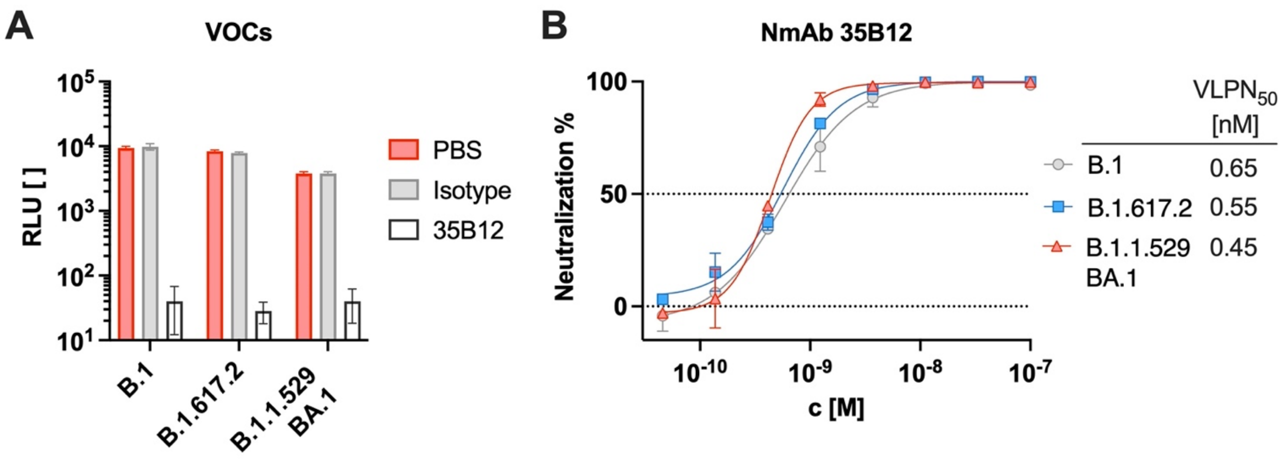

Supplementary Figure S3. Experiments with SARS-CoV-2 Spike variants.

(A) S^+ VLPs that comprise SARS-CoV-2 B.1 spike or its variants of concern (VOC) B.1.617.2 Delta or B.1.1.529 BA.1 Omicron were generated. Preparations were pre-incubated with 100 nM NmAb 35B12, IgG isotype control or diluent PBS, mixed with U251MG derived $ACE2^+$ EV (CD63~$LgBiT^+$) and incubated at 37°C for 4 h prior to addition of substrate and luminescence analysis (RLU).

(B) B.1, B.1.617.2 Delta or B.1.1.529 BA.1 Omicron variants of S^+ VLPs were pre-incubated with various concentrations (c [M]) of S specific NmAb 35B12. Next, samples were mixed with $ACE2^+$ acceptor EVs, incubated, analyzed and normalized as described for the cfVLPNT. Antibody concentrations for 50% VLP neutralization ($VLPN_{50}$) are indicated.

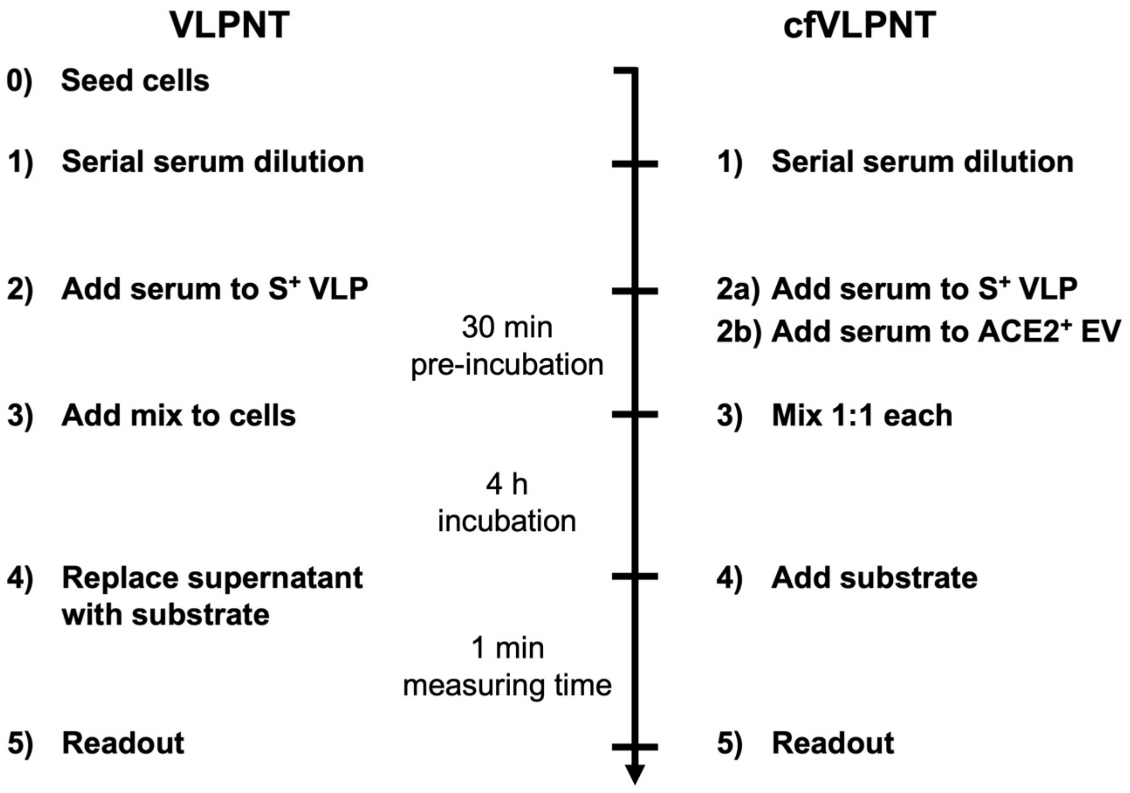

Supplementary Figure S4. Comparison of cell-based and cell-free VLP neutralization tests.

A schematic comparison of the cell-based VLP neutralization test (VLPNT) and the cell free VLPNT (cfVLPNT) at the level of single working steps is shown. While both diagnostic tests require qualified S^+ VLP preparations and sequential serum dilutions, the protocol of the cfVLPNT does not require prior seeding of $ACE2^+$ U251MG cells but uses $ACE2^+$ EVs instead. The cfVLPNT approach requires little preparation and can be conducted with frozen reagents.

Supplementary Table S1. Individual data of analyzed human serum samples.

	ID	Sex	Age	Sampling	COVID-19 severity	RT-qPCR	Vaccination scheme: Prime	Vaccination scheme: Boost	d p. PCR[a]	d p. Boost[b]	VLPNT B.1: VLPN$_{50}$ [1:]	VLPNT B.1: Titer$_{50}$[c] [IU/mL]	cfVLPNT B.1: VLPN$_{50}$ [1:]	cVNT B.1: VNT$_{100}$[d] [1:]	cVNT B.1: Titer$_{100}$ [IU/mL]
COVID-19 patients	C01	f	71	Apr 2020	moderate dis.	+			17		143	222	658	161	427
	C02	f	69	Apr 2020	critical dis.	+			28		62	96	214	†4	†11
	C03	f	69	Apr 2020	critical dis.	+			30		60	93	214	†4	†11
	C04	f	31	Apr 2020	mild dis.	+			50		42	65	72	†4	†11
	C05	m	62	Apr 2020	critical dis.	+			15		21	33	30	8	21
	C06	m	36	May 2020	mild dis.	+			21		98	152	357	81	215
	C07	m	54	May 2020	moderate dis.	+			23		857	1329	3215	≥1024	≥2716
	C08	f	67	May 2020	severe dis.	+			30		62	96	104	20	53
	C09	f	69	May 2020	critical dis.	+			48		20	31	91	†4	†11
	C10	m	56	May 2020	moderate dis.	+			44		42	65	49	10	27
	C11	m	59	Dec 2020	critical dis.	+			38		57	88	96	†4	†11
	C12	m	58	Dec 2020	critical dis.	+			15		15	23	87	†4	†11
	C13	m	77	Nov 2020	severe dis.	+			18		207	321	464	128	340
	C14	m	47	Dec 2020	severe dis.	+			15		1250	1938	5587	≥1024	≥2716
	C15	f	59	Dec 2020	moderate dis.	+			49		41	64	63	8	21
	C16	m	88	May 2020	severe dis.	+			30		299	464	769	256	679
	C17	m	56	May 2020	moderate dis.	+			47		48	74	99	10	27
	C18	m	32	May 2020	severe dis.	+			14		33	51	128	†4	†11
	C19	m	43	May 2020	critical dis.	+			22		44	68	76	16	42
	C20	m	88	May 2020	severe dis.	+			37		341	529	908	256	679
	C21	m	56	May 2020	moderate dis.	+			51		68	105	109	10	27
	C22	m	56	May 2020	moderate dis.	+			54		77	119	140	16	42
	C23	m	32	May 2020	severe dis.	+			21		1035	1605	2258	323	857
	C24	m	81	May 2020	critical dis.	+			50		341	529	1351	406	1077
	C25	f	56	Dec 2020	mild dis.	+			43		224	347	614	102	271
	C26	m	58	Dec 2020	critical dis.	+			26		116	180	154	†4	†11
	C27	f	68	Dec 2020	moderate dis.	+			27		15	23	56	†4	†11
	C28	f	63	Jun 2020	critical dis.	+			18		60	93	185	20	53
	C29	f	72	Jun 2020	critical dis.	+			25		35	54	30	8	21
	C30	m	72	Jun 2020	critical dis.	+			43		500	775	1080	20	53
	C31	m	43	Jun 2020	critical dis.	+			47		237	367	1408	203	538
	C32	m	72	Jun 2020	critical dis.	+			47		303	470	1020	16	42
	C33	m	23	Jun 2020	moderate dis.	+			49		93	144	188	51	135
	C34	m	43	Jun 2020	critical dis.	+			54		32	50	65	8	21
	C35	m	72	Jun 2020	critical dis.	+			54		263	408	980	10	27
	C36	m	43	Jun 2020	critical dis.	+			35		168	260	688	128	340
	C37	m	72	Jun 2020	critical dis.	+			35		276	428	741	25	66
	C38	m	43	Jul 2020	severe dis.	+			19		1112	1724	6863	≥1024	≥2716
	C39	m	59	Nov 2020	severe dis.	+			14		206	319	505	81	215
	C40	m	76	Nov 2020	severe dis.	+			16		528	819	2506	512	1358
	C41	m	36	May 20	mild dis.	+			44		63	98	168	32	85
	C42	m	43	May 20	critical dis.	+			27		41	64	277	128	340
	C43	m	72	May 20	critical dis.	+			28		38	59	342	10	27
	C44	f	89	Aug 20	moderate dis.	+			15		101	157	295	81	215
	C45	m	68	Sep 20	severe dis.	+			16		>2000	>3101	5263	≥1024	≥2716
	C46	m	31	Sep 20	critical dis.	+			18		1302	2019	2273	813	2156
	C47	f	77	Oct 20	critical dis.	+			22		546	847	2258	645	1711
	C48	f	76	Oct 20	severe dis.	+			20		60	93	153	10	27
	C49	m	82	Oct 20	critical dis.	+			23		165	256	568	128	340
	C50	f	78	Nov 20	critical dis.	+			15		35	54	127	13	34
	C51	f	76	Oct 20	severe dis.	+			23		381	591	1639	645	1711
	C52	f	77	Oct 20	critical dis.	+			36		320	496	1408	512	1358
	C53	f	61	Apr 20	severe dis.	+			16		330	512	1351	645	1711
	C54	m	77	Apr 20	severe dis.	+			17		413	640	752	203	538
	C55	m	77	Apr 20	severe dis.	+			21		732	1135	1266	256	679
	C56	m	88	May 20	severe dis.	+			22		470	729	926	256	679
	C57	f	62	Jun 20	moderate dis.	+			47		197	305	806	161	427

Continued on next page

							Vaccination scheme				VLPNT B.1		cfVLPNT B.1	cVNT B.1	
	ID	Sex	Age	Sampling	COVID-19 severity	RT-qPCR	Prime	Boost	d p. PCR[a]	d p. Boost[b]	VLPN$_{50}$ [1:]	Titer$_{50}$[c] [IU/mL]	VLPN$_{50}$ [1:]	VNT$_{100}$[d] [1:]	Titer$_{100}$ [IU/mL]
COVID-19 vaccinees	S001	f	93	Feb 2021			BNT162b2	BNT162b2		24	100	155	299		
	S005	m	26	May 2021			AZD1222	BNT162b2		13	82	113	769		
	S008	m	60	May 2021			AZD1222	BNT162b2		14	27	73	465		
	S011	f	28	Jul 2021			mRNA-1273	mRNA-1273		19	352	860	2309		
	S012	m	66	Jul 2021			AZD1222	mRNA-1273		15	99	242	1316		
	S014	m	70	Jul 2021			AZD1222	AZD1222		37	63	154	833		
	S015	f	32	Jul 2021			mRNA-1273	mRNA-1273		13	267	654	3571		
	S016	f	59	Jul 2021			BNT162b2	BNT162b2		56	38	91	323		
	S025	f	68	Aug 2021			AZD1222	mRNA-1273		22	254	697	2849		
	S026	m	66	Aug 2021			AZD1222	mRNA-1273		15	92	162	778		
	S027	f	58	Aug 2021			BNT162b2	BNT162b2		14	267	734	1539		
	S033	f	60	Sep 2021			BNT162b2	BNT162b2		29	69	162	297		
	S034	m	59	Sep 2021			BNT162b2	BNT162b2		90	40	94	394		
Healthy, naive donors	S003	m	24	Aug 2019							5	8	126		
	S006	m	56	Oct 2017							14	22	106		
	S009	f	26	May 2019							†0	†0	102		
	S018	f	30	Oct 2017							16	25	59		
	S019	f	69	Nov 2017							†0	†0	137		
	S020	f	35	Nov 2017							†0	†0	106		
	S021	f	43	Dec 2017							†0	†0	86		
	S022	f	53	Dec 2017							†0	†0	61		
	S023	f	31	Oct 2017							†0	†0	74		
	S024	m	na	Dec 2017							6	9	62		
	S028	m	30	Mar 2019							†0	†0	†0		
	S029	f	32	Mar 2019							10	16	†0		

m, male; f, female; na, not available; dis., disease; d, days; p., post; †, below LOD
[a]Days between first positive SARS-CoV-2 RT-qPCR report and sampling
[b]Days between booster dose and sampling
[c]VLPN$_{50}$ titer normalized to the WHO reference serum NIBSC 20/136 (1000 IU/mL)
[d]Reciprocal geometric mean titer from the highest serum dilution displaying 100% reduction of CPE based on three replicates
